# Stroke impairs the control of isometric forces and muscle activations in the ipsilesional arm

**DOI:** 10.1038/s41598-021-96329-0

**Published:** 2021-09-17

**Authors:** Laura Pellegrino, Martina Coscia, Psiche Giannoni, Lucio Marinelli, Maura Casadio

**Affiliations:** 1 Dept. Informatics, Bioengineering, Robotics and Systems Engineering, University of Genoa, Via Opera Pia 13, 16145 Genoa, Italy; 2 Bertarelli Foundation Chair in Translational Neuroengineering, Ecole Polytechnique Federale de Lausanne, Lausanne, Switzerland; 3 Wyss Center for Bio- and Neuroengineering, Geneva, Switzerland; 4 Division of Clinical Neurophysiology, Department of Neuroscience, IRCCS Ospedale Policlinico San Martino, Genoa, Italy; 5 Department of Neuroscience, Rehabilitation, Ophthalmology, Genetics, Maternal and Child Health, University of Genoa, Genoa, Italy

**Keywords:** Motor control, Stroke

## Abstract

Stroke often impairs the control of the contralesional arm, thus most survivors rely on the ipsilesional arm to perform daily living activities that require an efficient control of movements and forces. Whereas the ipsilesional arm is often called ‘unaffected’ or ‘unimpaired’, several studies suggested that during dynamic tasks its kinematics and joint torques are altered. Is stroke also affecting the ability of the ipsilesional arm to produce isometric force, as when pushing or pulling a handle? Here, we address this question by analyzing behavioral performance and muscles’ activity when subjects applied an isometric force of 10 N in eight coplanar directions. We found that stroke affected the ability to apply well-controlled isometric forces with the ipsilesional arm, although to a minor extent compared to the contralesional arm. The spinal maps, the analysis of single muscle activities and the organization of muscle synergies highlighted that this effect was mainly associated with abnormal activity of proximal muscles with respect to matched controls, especially when pushing or pulling in lateral directions.

## Introduction

Daily living activities such as opening a door, writing with a pencil, manipulating common objects, require efficient control not only of upper limb movements, but also of contact forces. Several stroke survivors suffer from a severe impairment of the contralesional arm and rely mostly on the ipsilesional arm to perform daily living activities.

With this often called ‘not impaired’ or ‘not affected’ arm, they are able to perform several tasks that before the stroke event they were performing with the contralesional arm or bimanually, undergoing a re-learning process that leads to a functional reorganization of their upper body motions and of their force control strategies^1–4^. However, while several studies investigated extensively motor and force deficits in the contralesional arm, the studies related to the ipsilesional arm are focused mainly on kinematics alterations^5–10^. Few studies suggested that each brain hemisphere contributes not only to the control of the movements of the contralesional side of the body, but also to the ipsilesional side, thus a lesion in one hemisphere affects the motor control of both sides of the body^5^. Interestingly, these works attributed the deficits related to dynamic control to a difficulty in modulating torque as movement amplitude increased^5,8^.

However, despite its importance, the ability after stroke to apply contact forces with the ipsilesional arm and its neural correlates at the level of muscle activity has not been consistently investigated in relation to single muscle deficits^11^ or to the ability to coordinate the activity of multiple muscles acting together (e.g. muscle synergies). While motions and forces are controlled concurrently in tasks involving mechanical interactions with the external environment they likely have separate neural representations^12–17^. Thus, an investigation that specifically focuses on force control tasks could lead to new insights on the impairments induced by stroke and on the consequent functional reorganization, supporting new rehabilitation protocols and enhancing the recovery process.

As a first step toward this goal, we investigated a pure force control task, eliminating confounds due to motion and dynamics effects, and we focused on a reaching task in isometric conditions^18,19^. In this context, Roh and colleagues^19^ proposed an isometric protocol to match task variables across control and stroke-impaired subjects and investigated muscle synergies. They examined the spatial activation patterns of elbow and shoulder muscles in the affected arm of moderate to severe stroke subjects and in both arms of age-matched controls. Muscle synergies involving proximal muscles exhibited consistent alterations following stroke: the anterior deltoid was co-activated with medial and posterior deltoids within the shoulder abductor/extensor synergy, and the shoulder adductor/flexor synergy in stroke was dominated by activation of pectoralis major, with limited anterior deltoid activation. Recruitment of the altered shoulder muscle synergies was strongly associated with abnormal task performance. Their results suggested that a simple isometric task could highlight clinically relevant features in post-stroke subjects, such as impaired control of the individual deltoid heads among the causes of post-stroke deficits in arm function. Unfortunately, only the contralesional arm of the stroke survivors was studied limiting the full characterization of upper limb deficits post-stroke during an isometric task.

Here, we systematically characterized behavioral performance, muscle activity and coordination, focusing on spinal maps and muscle synergies during an isometric force task performed with the upper limb by chronic stroke subjects, comparing them with aged-matched controls. We tested the two arms separately in consecutive sessions, not concurrently. The hand applied forces in different coplanar directions, as the forearms rested on a table and suitable holders prevented the motion of arm and wrist. The upper limb was supported against gravity performing actions feasible also by for the most impaired individuals. Thus, we used a simplified task with respect to Roh and colleagues^19^. Recently we proposed a similar assessment for people with multiple sclerosis^20^, finding that the exertion of isometric forces highlighted clinically meaningful alterations in behavioral parameters, muscle activity and synergies better than the execution of movement trajectories in different dynamical environments.

In addition to muscle synergies analysis already proposed by Roh and colleagues^19^, to analyze the whole multidimensional EMG activity, we estimated the spatiotemporal activity of motoneuronal pools belonging to the spinal cord, also named “spinal map”. This analysis has been introduced as a possible tool to describe the output activity of spinal cord circuits to the muscles. Spinal maps represent the spatiotemporal organization of the EMG signals and are a useful and simplifying tool to explore muscle organization, especially in multidimensional EMG analysis^21–27^.

To the best of our knowledge, the spinal maps were never used as a tool to represent muscle activity during a force control task in both unimpaired and stroke individuals.

The results identify specific features in behavioral performance, spinal maps and muscle synergies that are affected by stroke. More importantly, we found that, despite the force generation in the contralesional arm being more impaired, the ipsilesional arm is also affected as it is dissimilar to controls both in the behavior and in muscle activities.

## Results

### Overview

We investigated motor performance and muscle activity in 30 subjects during an isometric force task.

Fifteen were chronic stroke survivors (S, 10 females, F; 61 ± 10 years), and fifteen were their age and gender-matched unimpaired controls (C, 10 female, 60 ± 10 years, see supplementary material Table ST I). Subjects controlled the movement of a cursor on the screen applying isometric forces with their hand.

**Figure 1:**
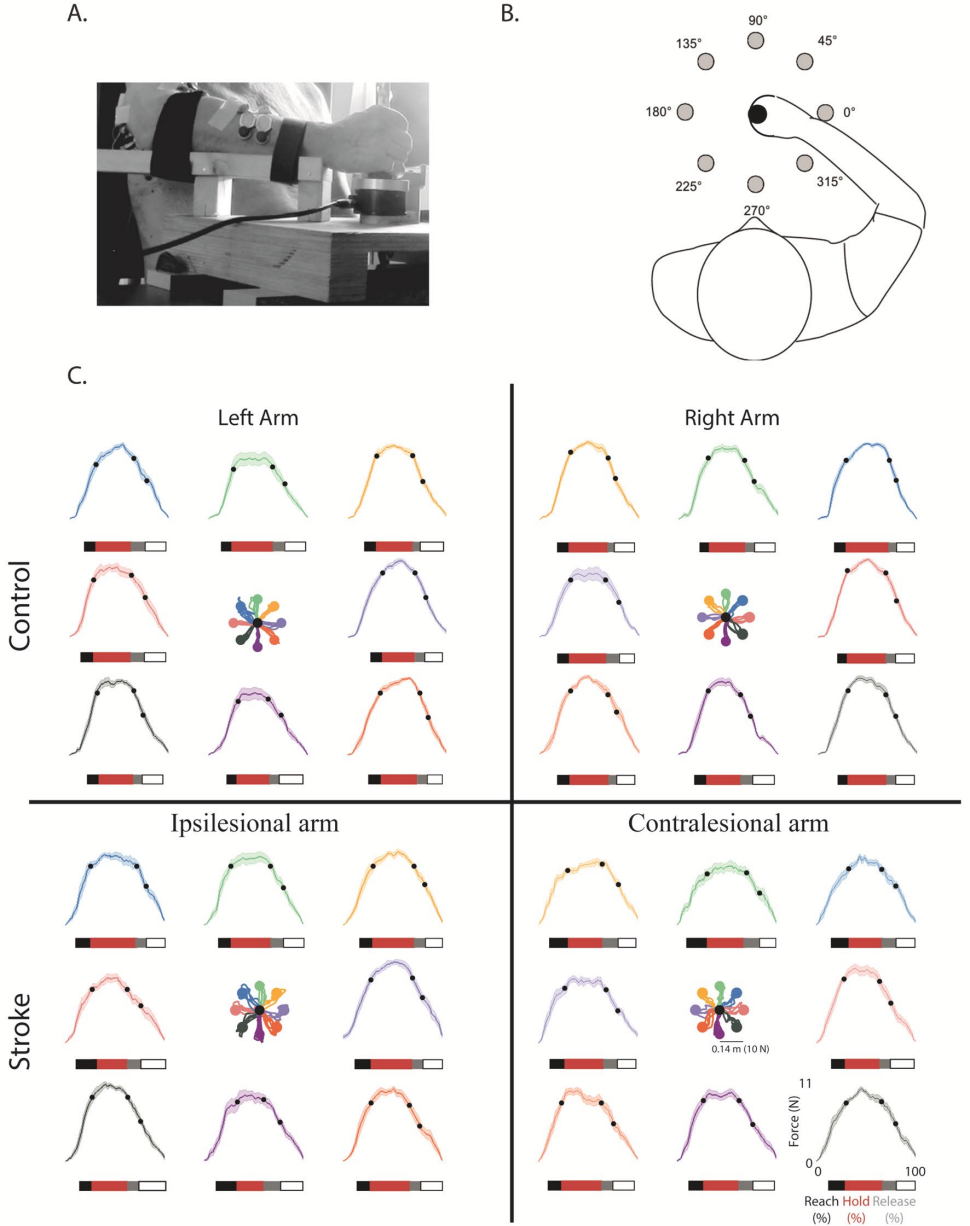
Top Panels: Experimental set-up. Subjects sat in front of a computer screen holding a force sensor with their forearm resting on a custom-made support that prevented their motion (**A**). Subjects controlled the movement of a cursor on the screen applying isometric forces with their hand. They had to reach targets at 14 cm distance, corresponding to 10 N force, in 8 different directions (**B**). Bottom panel (**C**): Cursor trajectories and force profiles for the left (first column) and by right (second column) hand of a control subject (first row), and for the ipsilesional and contralesional hand of a stroke subject (second row). We considered equivalent directions that corresponded in joint coordinates and the left-arm forces (and corresponding cursor trajectories) were mirrored at the midline in the endpoint space. Colors indicate equal directions in the endpoint space. The black dots on each force profile represent the transition points between each phase of the force exertion (cursor movement). The colors of the bars below each profile referred to these phases: (black) reaching phase where the applied force increased from 0 to 10 N corresponding to the center-out movement of the cursor; (red) holding phase where that reached force level was maintained corresponding to keeping the cursor inside the target; (gray and white) force release—grey the force decrease from 10 to 5 N, white from 5 to 0 N corresponding to the out-center movement of the cursor.

They had to reach eight equi-spaced targets presented one at the time on an imaginary circle (Fig. 1B) at 14 cm distance from the central position, corresponding to a force of 10 N (scale factor: 1 N force = 1.4 cm cursor displacement). Specifically, subjects were instructed to apply 10 N force reaching with the cursor (controlled by the applied force) the current peripheral target, to hold this force for 5 s, then release the force, i.e., go back with the cursor to the central target (corresponding to 0 N force). Thus, the center-out, holding, and out-center cursor movements were part of the same movement trial. Subjects were asked to reach the targets as accurately as possible, without time constraints, thus they performed the task at their self-selected speed. Subjects performed the tasks with both arms: stroke subjects always started with their contralesional arm, and their matched controls started with the same arm.

Surface EMG signals were recorded during task execution from the following 16 muscles: Triceps Brachii long head (TB-long), triceps Brachii lateral head (TB-lat), Biceps Brachii short head (BB-short), Biceps Brachii long head (BB-long), brachioradialis (BRAD), pronator teres (PRON), infraspinatus (INFR), latissimus dorsi (LAT), upper trapezius (TRAP), rhomboid major (RHOM), pectoralis major (PECT), anterior deltoid (DELT-ant), medial deltoid (DELT-mid), posterior deltoid (DELT-post), extensor carpi radialis (EXTE) and flexor carpi radialis (FLEX).

Muscle activity was investigated with three different analyses: spinal maps, single muscle and muscle synergies activity. All these analyses are performed starting from the same EMG signals, but they can be used to highlight different aspects related to muscle activity and can provide complementary information. Indeed, in multidimensional analysis of EMG signals (16 muscles for each arm) spinal maps are a useful tool providing an overview of the muscle activity and detecting in which muscles groups (e.g., proximal, distal arm muscles) there are alterations. This is also a starting point for a deeper investigation aiming at understanding which muscle features, and/or which group of muscles synergistically active, are altered. The first information is provided by single muscle analysis and the latter by the muscle synergy analysis. Muscle synergies, obtained from the linear factorization of the EMG envelopes, are suggested not only to describe which muscles are activated and how, but also to reveal how the CNS synergistically controls groups of muscles^28^.

When comparing the two arms, we considered equivalent directions that corresponded in joint coordinates. In the endpoint space, the left-arm forces and the directions of action of muscle activities and synergies were mirrored at the midline to be compared with the corresponding right-arm forces and muscle directions. Thus, the target directions indicated in the following text and figures as 315°, 0°, 45° corresponded to rightward forces of the right arm and leftward forces of the left arm, viceversa the target directions 135°, 180°, 225° corresponded to leftward forces of the right arm and rightward forces of the left arm.

### Force trajectories were slower, less smooth and less accurate in stroke subjects in both the contralesional and the ipsilesional arm, although the former presented higher deficits

Stroke subjects had worse behavioral performance than controls. Their force profiles were less smooth and more irregular, both in the contralesional and ipsilesional arm (Fig. 1C, a table of the indicators is reported in Supplementary Materials Table ST IV).

**Figure 2:**
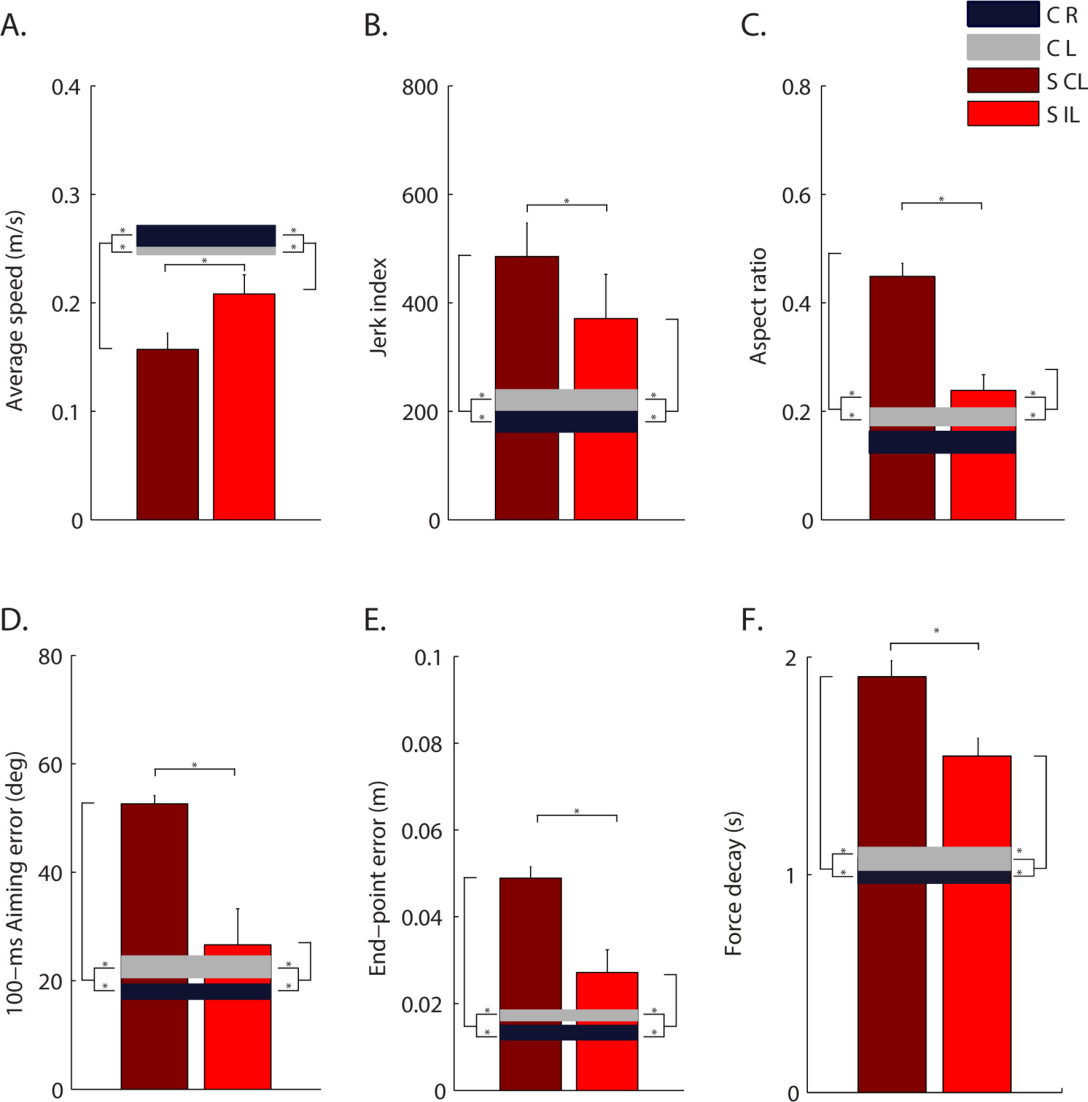
Features of the isometric force applied by the subjects to control the cursor trajectories (1.4 cm = 1 N). Control subjects (C) and stroke subjects (S) are shown with different colors as indicated in the legend. Darker and brighter colors represent the right (R) and left (L) arm respectively in the control subjects, and contralesional (CL) and ipsilesional (IL) arm in the stroke subjects. The error bars indicate the standard error of the indicators. * indicates significant differences (p < 0.05). The indicators (**A**–**E**) are related to the center-out movements not including the holding time. Indicator F is related to the force release phase. (**B**,**C**) The indicators jerk index and aspect ratio are dimensionless.

The cursor trajectories controlled by their forces had lower average speed (disease effect—F(1,28) = 22.68, p < 0.001), lower smoothness (jerk index: F(1,28) = 10.07, p = 0.004) and were also less accurate with a larger aspect ratio (F(1,28) = 12.99, p = 0.001) and greater end-point errors (F(1,28) = 16.01, p < 0.001). Also the directional errors at the beginning of the force exertion were higher (100-ms aiming error: F(1,28) = 18.99, p < 0.001); Fig. 2. The time required for the force to decrease by 5 N, i.e. half of the target value, was higher (force decay: F(1,28) = 14.71, p = 0.001); see Fig. 2F.

As expected, the contralesional arm had worse performance than the ipsilesional arm (disease $X$ arm effect—average speed: F(1,28) = 5.52, p = 0.026; jerk index: F(1,28) = 4.76, p = 0.038, aspect ratio: F(1,28) = 7.76, p = 0.009; 100-ms aiming error: F(1,28) = 12.15, p = 0.002; end-point error: F(1,28) = 6.42, p = 0.017 and force decay: F(1,28) = 5.49, p = 0.012); Fig. 2.

However, we observed a significant difference also between the ipsilesional arm of stroke subjects and the corresponding arm of controls in all indicators, i.e., lower average speed (p = 0.001), higher jerk (p = 0.001) 100-ms aiming error (p < 0.001), end-point error (p = 0.001), aspect ratio (p = 0.042), and force decay (p = 0.001).

### Spinal maps were altered in the contralesional and the ipsilesional upper limb

**Figure 3:**
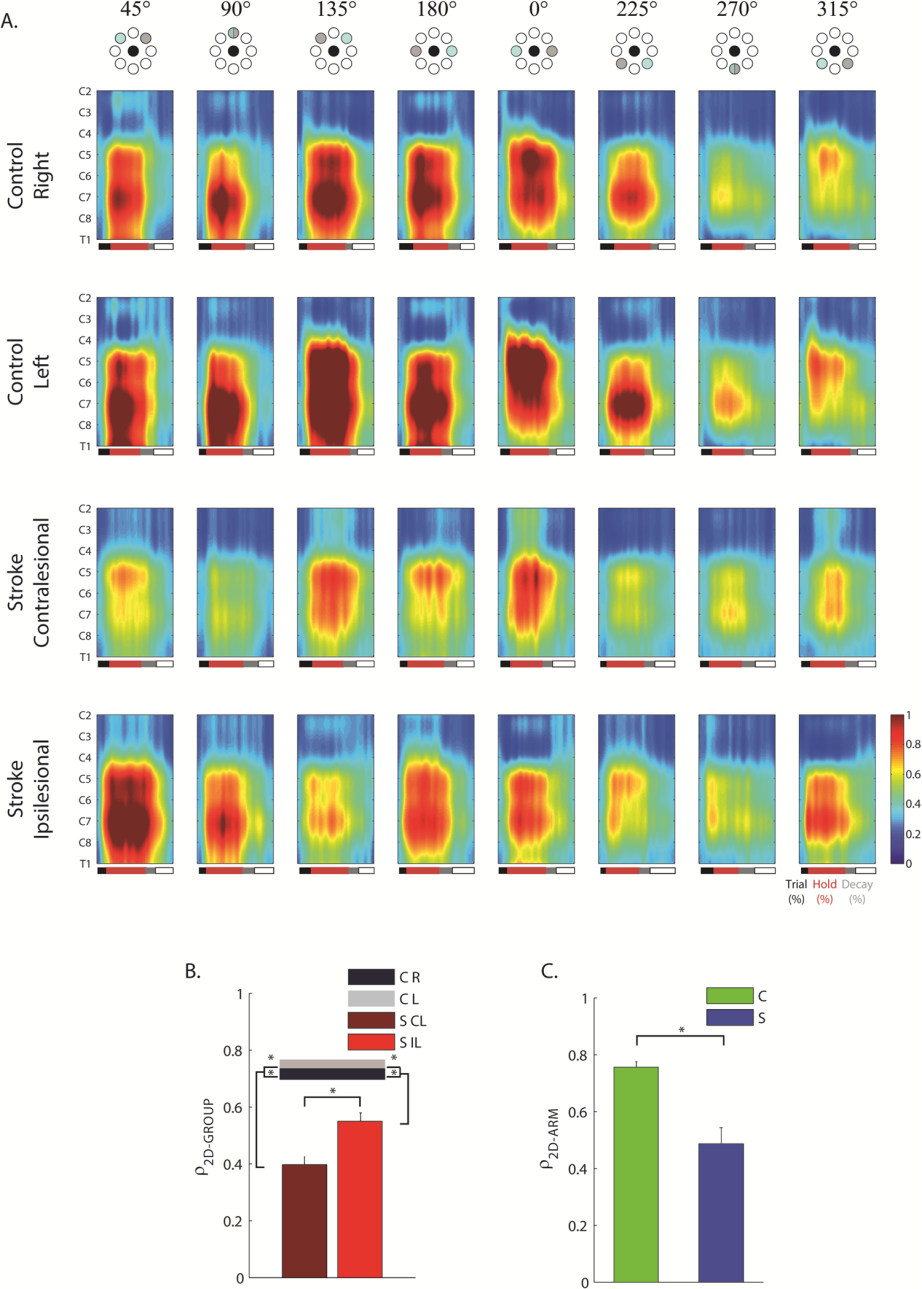
The spinal maps and correlation coefficients of muscle activations. (**A**) The spinal maps during force exertion in the eight directions for the right (first row) and left (second row) arm of a control subject (C) as well as the contralesional (third row) and ipsilesional (fourth row) arm of a stroke subject (S). On the x-axis the duration of the movement is represented in percentage of the total duration. Relative amplitude is denoted by a color scale (right calibration bar, see supplementary material ST II—for more details). Bars to the bottom of maps denote trial-to-release transition time. Muscle activations are referred to equal directions in the joint space; i.e. for each column the top panel indicates the corresponding target directions (cyan target) for the right arm, while the corresponding target directions of the left arm were mirror symmetric with respect to the vertical midline. (**B**) Mean and standard error of the ρ_2D-INTER-GROUP_ related to the spinal map similarity between control (C) and stroke subject (S) both for contralesional (CL, dark color) and ipsilesional (IL, light color) arm. The black (right arm) and grey (left arm) bars indicate the intra-group similarity (ρ_2D-INTRA-GROUP_), in terms of ρ_2D_, between the spinal maps of the control (C) group. Panel C: Mean and standard error of the ρ_2D-ARM_ related to the similarity between right and left arm of the control subjects (C; green bar) and between contralesional and ipsilesional arm of stroke subjects (S; blue bar). The error bars indicate the standard error of the indicators. * indicates significant differences (p < 0.05).

Spinal maps allow the overview of multiple muscle activations as a projection of their activity on the spinal segments. In control subjects, the spinal maps were characterized by a main activation for 20% to 100% of the time of the force exertion. This activity was observed between C5 and T1 around 0°, 90°, and 135° and between C5 and C8 around 180°; see Fig. 3A.

The spinal map in stroke subjects differed from those of the control subjects in both arms, as reported by the 2D Pearson’s correlation coefficient (ρ_2D_; see Fig. 3B,C; comparing the ρ_2D-INTRA-GROUPS_ and the ρ_2D-INTER-GROUP_ disease effect: F(1,28) = 116.3, p < 0.001). This difference was mainly due to the prolonged main burst of the spinal map activity for stroke subjects at the end of the force exertion, while in the controls this activation decreased in amplitude toward the end of the trial especially around the directions 45°, 135°, and 180°. This was observable in both the contralesional and ipsilesional arm. However, as expected the difference between spinal maps was arm-dependent (disease $X$ arm effect: F(1,28) = 5.20, p = 0.032; Fig. 3B). In the contralesional arm of stroke subjects, the spinal map differed from those of controls, especially around the directions 0°, 45° and 315° (disease $\times$ arm $\times$ direction effect: p < 0.001) where an anomalous increased activity was extended towards C2, C3, and C4 (Fig. 3A). These spinal segments principally innervate TRAP, INFRA, and RHOM (see supplementary material Table ST II). This modulation of the spinal activity differed from those of the controls also in the ipsilesional arm, although to a lesser extent than in contralesional arm (Fig. 3B).

### Rhomboid and Trapezius were abnormally activated both in the contralesional and the ipsilesional arm

**Figure 4:**
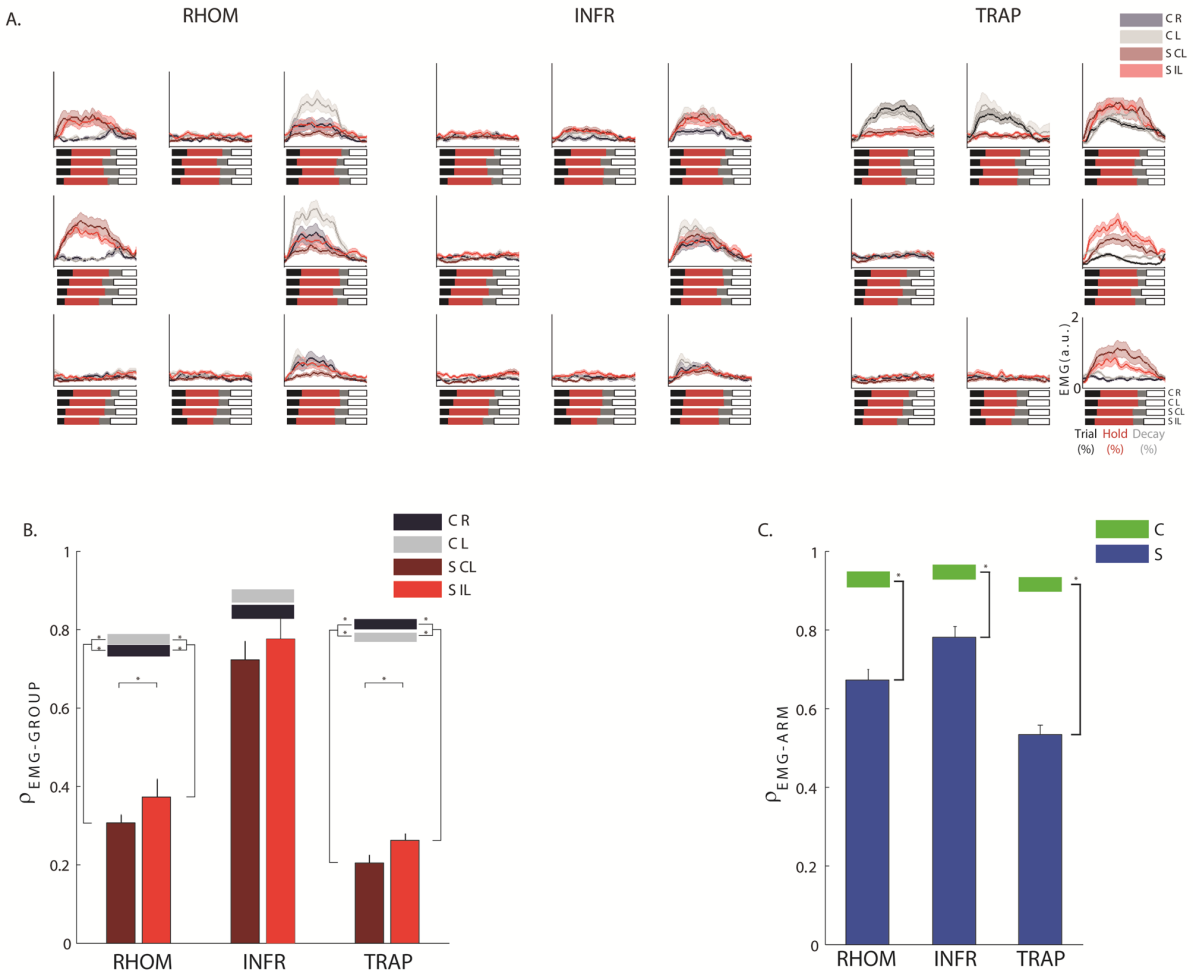
Top panels: Normalized EMG envelopes. Activation of the rhomboid major (RHOM, first column), Infraspinatus (INFR, second column) and upper trapezius (TRAP, third column) in the eight directions. The blank and red line respectively represents the mean activation for control subjects (C) and stroke subjects (S). Darker and brighter colors represent the right (R) and left (L) arm of C and contralesional (CL) and ipsilesional (IL) arm of S. The shaded area indicates the standard error. Bars to the bottom of EMG envelopes denote trial-to-release transition time. Bottom panels: Mean and standard error of the Pearson correlation comparing the EMG envelopes of the two groups (ρ_EMG-INTER-GROUP_, **B**) and the arms (ρ _EMG-ARM_, **C**). The darker and lighter colors in the B panel represent respectively the contralesional (CL) and ipsilesional (IL) arm for stroke subjects (S). The black (right arm) and grey (left arm) bars indicate the intra-group similarity (ρ _EMG-INTRA-GROUP_, **B**), in terms of ρ_2D_, between the EMG envelopes of the control (C) group. The blue and green colors in the (**C**) panel represent respectively the control (C) and stroke subjects (S). The error bars indicate the standard error of the indicators. * indicates significant differences (p < 0.05).

We investigated the activity of the single muscles comparing the two populations and the two arms. For many muscles, stroke subjects had muscle activations in the contralesional and in ipsilesional arm similar to control subjects. However, in both arms their RHOM and TRAP had different amplitude modulation (disease effect: F(1,28) = 12.60, p < 0.001 and F(1,28) = 10.41, p < 0.001, respectively) with respect to that of control subjects (Fig. 4A,B) and this difference changed depending on the direction (disease $\times$ direction effect: F(7,196) = 16.45, p < 0.001 and F(7,196) = 21.36, p < 0.001).

Specifically, these muscles had a more marked and prolonged phasic activity instead of the expected tonic activity; i.e. they presented an EMG waveform with a burst of activity (phasic activity), instead than maintaining a certain level of activity for the duration of the movement (tonic activity) in specific directions^29–31^: the RHOM in the medial directions (*i.e.* 135° and 180°: post-hoc analysis: p < 0.001), when the arm was extended, the TRAP in the lateral directions (*i.e*. 0°, 45°, and 315°: post-hoc analysis: p < 0.001) when the arm was flexed. In summary, stroke subjects increased their phasic activity, i.e., they presented EMG waveforms modulated in time and amplitude^29^ during medio-lateral force generation (i.e. 0°, 45°, and 315°, 135° and 180°) for these muscles.

The muscle activations between the two arms of a same subject were more different in the stroke than in the control subjects for all muscles and in particular for the RHOM, INFR and TRAP (disease effect: F(1,28) = 22.34, p = 0.001; F(1,28) = 11.40, p = 0.010 and F(1,28) = 24.20, p < 0.001, respectively); see Fig. 4C. For RHOM and TRAP, the difference in EMG activity between the contralesional and ipsilesional arm changed depending on the direction (disease $\times$ direction effect: F(7,196) = 34.54, p = 0.001 and F(7,196) = 11.62, p = 0.001), reflecting the increase in phasic activation described above.

### Stroke subjects and controls had the same number of muscle synergies, but these synergies had different structure and activation profiles in both the contralesional and ipsilesional arm

We used the non-negative matrix factorization (NNMF) algorithm to extract muscle synergies from the EMG envelopes^32–34^. In this view, a muscle synergy represents a set of muscles, which are simultaneously activated by a single temporal command. The organization of a synergy is determined by the contribution (i.e., weight coefficient) of each muscle, as specified by the weight matrix W. Its activation profile is defined by the activation coefficients, specified by the matrix H. To extract the muscle synergies, computing their weight and activation coefficients, we considered all trials (i.e., all repetitions and directions). For this isometric task, four muscle synergies were extracted for each subject in both arms. There was not a statistically significant difference in the number of muscle synergies between the two populations (disease effect: F(1,28) = 4.02, p = 0.155) and between arms (disease $X$ arm effect: F(1,28) = 0.173, p = 0.631); see Supplementary material Fig. S1A. This result indicates that in this task for stroke subjects, the dimensionality of muscle synergies in the contralesional and ipsilesional arm was preserved, although in several subjects we observed a reduction in the average number of muscle synergies for the contralesional arm, mainly due to a lower cumulative variance for synergy 3 and 4; see supplementary material Table ST III. However, the cumulative variance was not significantly different between populations (disease effect: F(1,28) = 2.23, p = 0.38) and between the contralesional and ipsilesional arm (disease $X$ arm effect: F(1,28) = 2.47, p = 0.30 and F(1,28) = 3.10, p = 0.41, respectively); see supplementary material Fig. S1B.

### Weight coefficients of the muscle synergies (W)

**Figure 5:**
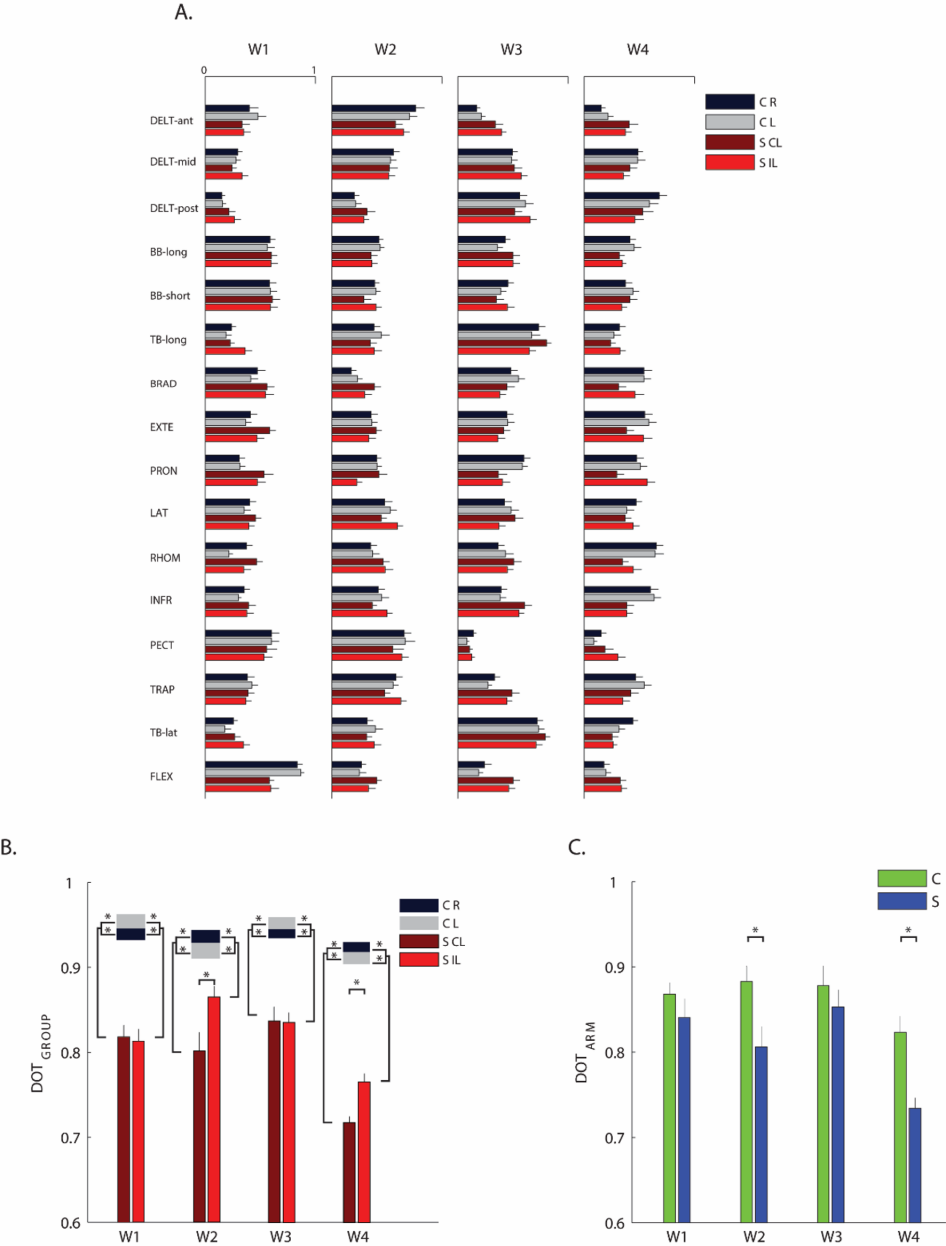
Weight coefficients of the muscle synergies. (**A**) Weight coefficients for the four muscle synergies (W1 to W4). Weight coefficients were computed considering all trials, i.e., including all directions and repetitions. Control subjects (C) and stroke subjects (S) are shown with different colors as indicated in the legend. Darker and brighter colors represent the right (R) and left (L) arm respectively for the control subjects and contralesional (CL) and ipsilesional (IL) arm in the stroke subjects. The error bars represent the standard error. (**B**) Comparison between groups (i.e., between stroke and control subjects) by the scalar product of weight coefficients of each muscle synergies (DOT_INTER-GROUP_). For the weight coefficient of each synergy, W1 to W4, darker and lighter red bars indicate the values obtained by comparing respectively the contralesional (CL) and ipsilesional (IL) arm of each stroke subject with the corresponding arm of his/her control subject, then averaging across the stroke group. The grey and black horizontal bars indicate the values obtained by comparing respectively the weight coefficients of the muscle synergies for the left (L) and the right (R) arm of one control subject with the corresponding arm of all the other controls, and then averaging across the control group (DOT_INTRA-GROUP_). The error bars correspond to the standard error. (**C**) Comparison between the two arms of a same subject by the scalar product of weight coefficients of the muscle synergies (DOT_ARM_). For the weight vector of each synergy, W1 to W4, blue and green bars indicated the values obtained by comparing the two arms of stroke subjects and controls, respectively. The error bars correspond to the standard error. (**B**,**C**): * indicates significant differences (p < 0.05) between stroke and control groups.

In both populations, the weighting coefficients represent the participation of each muscle into the synergies. We considered as main contributor to a synergy a muscle that by visual inspection presented a high weight coefficient for that synergy compared to (i) the weight coefficients of the same muscle in the other synergies and (ii) the weight coefficients of other muscles for the same synergy (Fig. 5A). Thus, organization of muscle synergies could be synthetically described as follows:

- Synergy 1 mainly involved the BB-long, BB-short and FLEX (Fig. 5A, W1).
- Synergy 2 mainly involved the DELT-ant, DELT-mid, LAT, PECT and TRAP (Fig. 5A, W2).
- Synergy 3 mainly involved the DELT-post, TB-lat, TB-long and PRON (Fig. 5, W3).
- Synergy 4 mainly involved the INFR, RHOM and EXTE (Fig. 5, W4).

To evaluate the similarity of the organization of each muscle synergy between stroke and control subjects, we used the DOT_GROUP_, a metric based on the scalar product between the weight coefficients’ vectors of two subjects (see methods for more details). This metric highlighted that the weight coefficients were different between stroke and control subjects (disease effect: F(1,28) = 103.5, p < 0.001). As expected, in the stroke subjects, this difference was larger for the contralesional than for the ipsilesional arm (disease $X$ arm effect: F(1,28) = 3.70, p = 0.032) and was synergy-dependent (disease $X$ synergies effect: F(3,84) = 3.60, p = 0.015); see Fig. 5B. Specifically, the weight coefficients of the proximal synergies (W2 and W4) differed more than those of the distal synergies (W1 and W3) when comparing controls and stroke survivors and also when comparing the ipsilesional and contralesional arm in the stroke population.

Notice that the two muscles that had different lateral activation in stroke subjects, the TRAP and the RHOM are among the main contributors to W2 and W4, respectively. When comparing contralesional and ipsilesional arm of stroke subjects (Fig. 5B), there was not significant difference in W1 and W3 (post-hoc analysis W1: p = 0.478 and W3: p = 0.894), while most of the muscles in W2 and W4 had a different contribution (post-hoc analysis: W2: p = 0.002 and W4: p < 0.001). For example, in W2 the TRAP, BB-short and LAT and in W4 the BRAD, EXTE, PRON, and RHOM had lower participation (weight coefficients) in the contralesional arm than in the ipsilesional arm, while the PRON in W2 had the opposite behavior.

This observation was also confirmed by the fact that W2 and W4 of stroke subjects showed less similarity between arms (DOT_ARM_) compared to controls (disease effect: F(1,28) = 7.05, p = 0.013, disease $X$ synergies effect: F(3,84) = 7.49, p = 0.040, post-hoc analysis: W2: p = 0.014 and W4: p < 0.001); Fig. 5C.

### Activation coefficients of the muscle synergies (H)

**Figure 6:**
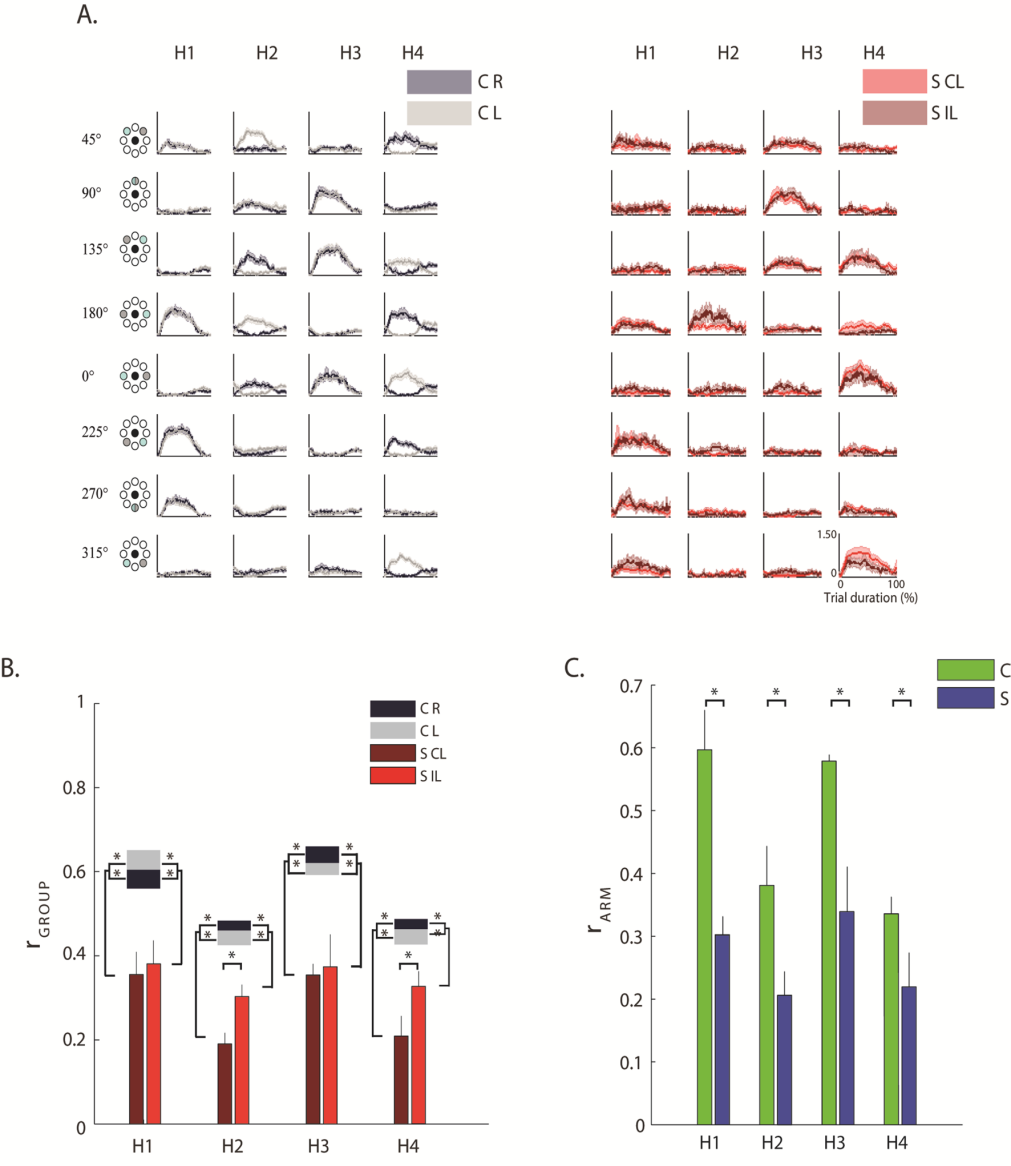
Activation coefficients of the muscle synergies. (**A**) The grey and red line represents the mean activation profiles of the four synergies (H1 to H4) for control subjects (C, on the left) and stroke subjects (S, on the right), respectively. Activation coefficients were computed considering all trials, i.e., including all directions and repetitions. Darker and brighter colors represent the right (R) and left (L) arm of C and contralesional (CL) and ipsilesional (IL) arm of S. The shaded area indicates the standard error. (**B**) Comparison between groups (i.e., between stroke and control subjects) by Pearson correlation (r) of the activation profile coefficients of the muscle synergies (r_INTER-GROUP_). For the activation coefficient of each synergy, H1 to H4, darker and lighter red bars indicated the values obtained by comparing respectively the contralesional (CL) and ipsilesional (IL) arm of each stroke subject with the corresponding arm of his/her control subject, then averaging across the stroke group. The grey and black horizontal bars indicate the values obtained by comparing respectively the activation profiles of the muscle synergies for left (L) and the right (R) arm of one control subject with the corresponding arm of all the other controls, and then averaging across the control group (r_INTRA-GROUP_). The error bars correspond to the standard error. (**C**) Comparison between the two arms of each subject by Pearson correlation (r) of the activation profile coefficients of the muscle synergies (r_ARM_). For the activation coefficients of each synergy, H1 to H4, blue bars indicated the average value obtained for the stroke subjects (S) and the green bars the average values obtained for the control subjects (C). The error bars correspond to the standard error. Panel B&C: * indicates significant differences between stroke and control groups.

In control subjects, the activation profile of each synergy was modulated across directions so that each synergy’s engagement was specific to one or two consecutive directions, and the activations of the whole set of muscle synergies allowed covering all the workspace (Fig. 6A). Specifically for the control subjects:

- Synergy 1 was mainly active for forces exerted toward targets in 180°, 225°, 270° directions (Fig. 6A, H1) for both arms.
- Synergy 2 was mainly active for forces exerted toward 180° and  45° for the left arm, with mirror-symmetric activations for the right arm, for control subjects, but toward 180° for stroke subjects (Fig. 6A, H2).
- Synergy 3 was mainly active for forces exerted toward targets in 0°, 135° and 90° directions (Fig. 6A, H3) for both arms.
- Synergy 4 was mainly active for forces exerted towards 180°, 45° and 225° for the right arm, with mirror-symmetric activations for the left arm, for controls, but toward 0°, 135° and 315° for stroke subjects (Fig. 6A, H4).

To evaluate the similarity of the activation profiles of each muscle synergy between stroke and control subjects we used the r _GROUP_, a metric based on the Pearson correlation between the activation profiles of two subjects (see methods for more details).

This metrics highlighted that in stroke subjects the profiles were significantly altered compared to controls (disease effect: (F(1,28) = 30.75, p < 0.001). These differences were observable when pushing or pulling in lateral directions, both in the contralesional and the ipsilesional arm, although, as expected, the contralesional arm had more marked alterations than the ipsilesional arm (disease $X$ arm effect: F(1,28) = 333.81, p = 0.022; Fig. 6B) especially for H2 and H4 (the activation coefficients of the muscle synergies including the RHOM and the TRAP among other muscles). This expected difference was confirmed by the low value of r_ARM_, as result of the direct comparison of the contralesional and ipsilesional arm of each stroke subjects, with respect to the same parameter obtained when comparing the two arms of the control subjects (disease effect: F(1,28) = 25.48, p < 0.001; Fig. 6C).

## Discussion and conclusion

This study aimed at characterizing muscles and force deficits in both arms by providing metrics sensitive to chronic stroke. There are three important elements of novelty:

1. The evaluation of the ability to apply isometric force by the ipsilesional hand, investigating both behavioral force performance and muscular activity;
2. The description in terms of spinal maps related to the generation of isometric forces in the upper limbs of both controls and stroke subjects;
3. A deeper investigation of the activity and synergies related to the proximal shoulder and back muscles such as the RHOM and the TRAP during isometric force tasks, that we found altered in stroke subjects, but were not recorded in previous similar studies also in the contralesional arm^18,19^.

We selected a simple isometric planar task with arm gravity compensation to propose an assessment that stroke subjects with different levels of impairment could complete with both arms.

Our results show that moderate to severe chronic stroke subjects generated less straight, smooth and accurate force profiles than controls in both the ipsilesional and contralesional arm, although in the contralesional the alteration was, as expected, higher. The spinal maps, the muscle synergies, and the analysis of single muscle activity provided congruent evidence of altered activity mainly of proximal muscles in the ipsilesional and contralesional arm comparing to controls.

Indeed, spinal maps evidenced that the altered spinal segments were mainly C2, C3, and C4: among the muscles shared by these 3 spinal segments there were TRAP, INFRA, and RHOM. From this, we expected to find main alterations in the EMG envelopes of those muscles and not in the other muscles. This is what we found with the EMG envelope analysis. The spinal map analysis helped us to synthetically show that for most muscles the activity was preserved, while the analysis of the EMG envelopes highlighted that stroke subjects increased their phasic activity during medio-lateral force generation (i.e., 0°, 45°, and 315°, 135° and 180°) for these proximal muscles, especially for the contralesional arm, highlighting also an increased and prolonged phasic activity compared to controls. In addition, muscle synergies evidenced that among our subjects, the stroke did not change the number of groups of muscle synergistically activated, that were four for both populations, but modified both the contribution of individual muscles to each synergy and their timing activation. Thus, muscle synergies activations and weight coefficients account for the differences observed in the single muscle activity and in the spinal maps, supporting the same conclusions. In addition, the results highlighted an integrity for most of our stroke subjects of the number of muscle groups that need to be synergistically activated to perform this task. In the following we discuss these findings in detail.

### Chronic stroke subjects have different strategies than controls for generating isometric forces at the hand not only in the contralesional, but also in the ipsilesional arm

Chronic stroke subjects had more difficulties than controls executing the isometric force task also with the ipsilesional arm, showing greater errors, and more corrections both in the initial and final part of the force trajectory, although the ipsilesional arm had better performance than the contralesional arm. Several studies extensively investigated how stroke subjects adopt different strategies to control the ipsilesional arm with respect to their contralesional arm, and also to the corresponding arm of their matched controls^5,35^ in dynamic tasks, such as reaching^5,8,36–39^.

However, how stroke subjects control isometric forces was less explored and specifically the ability to control forces with the arm ipsilesional was not investigated. Here, we provide new evidence supporting the hypothesis that the control of isometric forces was altered also in the ipsilesional arm. This was revealed by a simple isometric planar task with the arm supported against gravity.

### Spinal maps differed in the contralesional and ipsilesional arm with relevant alterations in C2, C3 and C4

Spinal maps represent the spatiotemporal organization of multiple EMG signals as a projection of their activity on the spinal segments^21,22,24,27,40^. The estimated motoneuronal activity provided by spinal maps from superficial EMG signals has been used in upper and lower limb motor tasks to assess the impact of different factors on the spinal cord activity in controls^21,22,24–27,40,41^ and neurological patients, including spinal cord injury^21,23^ and stroke^42^.

This model allows estimating the location and the dynamics of the spinal circuits eliciting muscle activation during a task, and it provides us with a tool for describing the activation of many muscles at once during task execution in the different conditions.

So far, no study has investigated spinal maps related to the generation of isometric forces in the upper limbs in unimpaired subjects or stroke survivors. We found that in controls, the spatiotemporal MN activity was characterized by a main period of activation between the 20% and the 100% of the force exertion between the spinal segments C5 and T1 around the 0°, 90°, and 135°directions, and between C5 and C8 around the 180° direction. Due to the difficulty to normalize the muscle activity for the maximum voluntary contraction in stroke subjects, it is not possible to compare the absolute level of intensity of the spinal maps (and also of the muscle activity and muscle synergies) between arms or groups, but the analysis focused on their topology and their variation in the time-observation window. There, stroke subjects seemed to maintain or even increase for both arms the spinal map activity toward the end of the movement, as they had difficulties and delays decreasing muscle activations once started.

The spinal activity in the contralesional arm differed from controls especially around 0° direction. We observed an anomalous increased activity extended towards C2, C3 and C4. These spinal segments principally innervate TRAP, INFRA, and RHOM. The results of the analysis of spinal maps were consistent with the observation of single muscle activity, which also highlighted a different (and possibly increased) phasic activity of the RHOM in the medial directions (90° and 135°) and of the TRAP in the lateral direction (0°) in both arms, but especially in the contralesional arm.

These results are similar to what has been observed in dynamic tasks for the elbow agonist and antagonist muscles, where the primary impairment is mainly due to a limited and prolonged recruitment of agonist contraction and delayed cessation of agonist contraction at the termination of movement^43^ and sometimes also during the extension phase^44^. The abnormal prolonged and augmented activity is not the only upper limb muscle alteration known in stroke patients^43–46^. A decreased activity of some muscles (such as the triceps^44^ or the deltoid posterior^46^) might also occur, together with the coupling of shoulder adduction and elbow flexion or shoulder abduction with elbow extension^47^. We did not observe these last two features maybe due to the chosen task, an isometric force generation with gravity compensation: it is indeed known that gravity compensation can mitigate the abnormal flexor motor synergy coupling elbow and shoulder flexion^47^.

### Stroke altered mainly the structure and the activations of the proximal muscle synergies in both the contralesional and ipsilesional arm, although to a different extent

In both controls and chronic stroke subjects, four muscle synergies were extracted: two “proximal” synergies including mainly muscles controlling the shoulder, and two “distal” synergies including mainly muscles controlling the elbow.

Muscle synergies gained clinical relevance because their integrity is considered a physiological marker of motor cortical damage^20,32,48–50^ and there is evidence that the organization of muscle synergies after stroke depends on the level of impairment and on the onset of the cerebrovascular accident^32,48,51^.

Roh et al.^19^ also investigated upper limb muscle synergies during isometric force generation. They proposed a 3D isometric task and focused on the contralesional limb. Similarly to us, they found no differences in the number of muscle synergies between groups. This result differs from the one of other studies investigating muscle synergies after stroke. In severe chronic post-stroke individuals, the number of muscle synergies correlates with spasticity, reduced walking speed, clinical and biomechanical measures of balance and walking functions, such as propulsion asymmetry, step length asymmetry^49^. They also correlated with Fugl-Meyer assessment^48^, even better than gait and balance functions. Note that all these studies focus on motion while our study as the one of Roh and colleagues^19^ investigated isometric force production. Taken together these findings suggest that the number of the muscle synergies is more frequently altered in movement tasks, but not isometric force exertion^19^. This difference could be also due to the compensation of gravity that was used in our and Roh and colleagues’ study. Indeed, Tropea et al.^52^ did not find differences in the number of muscle synergies between control and stroke subjects also during planar reaching movements with gravity compensation.

We found that stroke modified the structure and the activations of all the four muscle synergies for both arms, although to a different extent. The difference between the contralesional and ipsilesional arm of stroke subjects was more marked for the two synergies mainly involving the proximal muscles than for the other two involving the distal muscle.

Roh et al.^19^ observed an alteration of the structure of muscle synergies: they found alterations in muscle synergies related to the activation of shoulder muscles in the contralesional arm of severely impaired stroke survivors. This alteration was mainly due to the deltoid anterior muscle that was coactivated with medial and posterior deltoids within the shoulder abductor/extensor synergy, and the shoulder adductor/flexor synergy in stroke was dominated by activation of pectoralis major, with limited anterior deltoid activation. We did not find significant alterations of the deltoids, and this might be because the planarity of our task combined with anti-gravity support reduced the need for this compensation strategy. However, we found alterations of the TRAP and the RHOM, that Roh et al. did not record, supporting with new evidence the hypothesis of alterations of muscle synergies related to the activity of the proximal muscles in stroke survivors. Moreover, we observed this alteration also in the ipsilesional side.

As another similarity with the results of Roh and colleagues, we also did not observe an abnormal coupling of shoulder and elbow muscles within individual muscle synergies as found by^47,53,54^, indicating that this alteration is probably not evident in isometric force generation and/or in the presence of gravity compensation, two aspects that our and Roh study have in common.

We observed that most of the muscles in W2 and W4 had a different contribution when comparing contralesional and ipsilesional arm of stroke subjects and also when comparing the two subjects’ groups, while this difference was less marked in W1 and W3. For example, in W2 the TRAP, BB-short and LAT, and in W4 the BRAD, EXTE, PRON, and RHOM had a lower participation (weight coefficients) in the contralesional arm than in the ipsilesional arm, while the PRON in W2 had the opposite behavior. Moreover, the activation profiles both in the contralesional and the ipsilesional arm were altered in the stroke survivors when pushing or pulling in lateral directions, mainly in the synergies involving proximal muscles. Our results extend the findings of Roh et al., because we observed also alterations in the structure and in the activations of muscle synergies in the ipsilesional arm that they did not investigate.

Results related to muscle synergies were consistent with what found in the analysis of spinal maps and of single muscle activity, showing that our stroke subjects had an altered activity especially in the proximal muscles for the control of the shoulder and trunk when exerting forces in certain directions, and especially in the contralesional arm, not only as a prolonged augmented phasic activity, but also as coupling with the other synergistic muscles to generate isometric forces. Note that the alterations found in the ipsilesional (non-paretic) arm could be also caused by the alteration of the ipsilesional motor pathways^55^. Thus, future neurophysiological studies (e.g., through TMS) could verify this hypothesis investigating the correlation between by the integrity of the ipsilesional pathways and the degree of motor impairment in the ipsilesional limb.

### Implications for chronic stroke assessment and rehabilitation

Directional errors in the force trajectory as well as metrics related to the first and second time derivatives of the applied force are all sensitive metrics to motor impairment induced by a stroke while generating isometric forces in the contralesional and ipsilesional upper limb. Each stroke subject showed differences with respect to controls in all these features related to the contralesional arm. Moreover, these metrics allow discriminating the different levels of impairment between arms in all subjects. The similarity metrics computed between limbs as well as between populations both for the spinal maps and for the weight and the activation coefficients of synergy 2 and 4 are also a sensitive metric to motor impairment induced by stroke.

Our results identify metrics that can constitute a quantitative framework for the investigation and assessment of upper limb impairments in chronic stroke subjects. They can be measured during the execution of a simple task that every stroke survivor can perform, without the use of complex and expensive equipment. Such quantitative framework as the one here presented enable rehabilitation professionals to devise treatment plans based on the residual capacity of individual patients. In this sense, our results show that not only the ability to generate movements, but also isometric force control is impaired at the behavioral and muscular level in both arms post-stroke, therefore rehabilitative treatments should consider motor training with also force exertion at both hands and bimanual tasks. This study could also pave the way for new longitudinal studies where the evolution of muscle recruitment during the rehabilitation program is quantitatively evaluated.

### Limitations

Stroke patients constitute an extremely heterogeneous population, therefore a larger number of subjects with diverse motor impairments, lesion sizes and sites etc. is necessary to confirm the results of this study. We investigated correlations between the level of impairment of the stroke subjects as reported by the Fugle-Meyer Assessment (FMA) score and all the metrics, but we did not find a significant correlation. We acknowledge that this would have significantly increased the impact of our results, but this could be mainly due to the limited number of subjects with respect to the range of their impairments, and for this reason, the study should be extended to a larger cohort of subjects.

In the presented framework two additional points to investigate are (i) the relationship between the dominant hemisphere and the stroke hemisphere^5–7^ (ii) the influence of the specific location of the stroke lesion. However, also these points would require a larger population of both left- and right- hemiparetic stroke patients, while here our subjects’ groups were too small for this investigation. We also acknowledge that we adopted a self-selected speed rather than a fixed speed for force generation. Reaching speed has an impact on upper limb muscle synergies^29,56^, therefore we cannot exclude that the different time and speed in task execution for the two populations might have influenced our results. However, forcing control or stroke subjects to generate a force profile at a fixed speed would have intrinsically altered force generation. Moreover the results on muscle synergies were the same, as in^57^, when considering only holding phase where all subjects performed the same task with equal duration, suggesting that the reported results were not biased by the different trial duration (see supplementary materials for details, Figs. S2–S4).

The arm support had also an influence on upper limb muscle synergies^58^, but it is a relevant aid to allow the correct execution of the task also for the most impaired individuals and for this reason it has been adopted also in other studies^19^.

We also acknowledge that stroke subjects had more variability in task execution respect than controls, therefore the investigation of muscle activity, spinal maps and muscle synergies is also affected by this variability and differences. However, differences in task execution derive from differences in muscle activity and coordination and the two are strictly entertained. Forcing the subjects to execute the task identically would have altered the force generation in a natural way.

We adopted the normalization of the muscle activity by the median value. Several different normalizations have been adopted in studies investigating muscle synergies, for example some used the maximum voluntary contraction^26,58,59^, the peak value among conditions^49^, the median value across conditions^32^, the variance^48^. A study demonstrated that the type of normalization does not significantly alter the characteristics of the synergies extracted^60^. Normalizing for the maximum voluntary contraction would have allowed the comparison of EMG, spinal maps and muscle synergies amplitudes among subjects, but unfortunately it is a difficult measure to be performed in neurological patients. Our results suffer the limitation of the impossibility to perform this comparison that can be relevant to identify differences among controls and stroke subjects.

Finally, our analysis could be extended by including upper limb 2D and 3D force generation, with and without gravity effect, at different controlled speeds.

## Materials and methods

### Subjects

30 subjects participated in this study, fifteen were stroke survivors (S: 5 males (M)–10 females (F); 61 ± 10 years) and fifteen were subjects without neuromotor deficits (C: 5 M – 10 F; 60 ± 10 years) recruited to serve as gender and age-matched controls. Stroke subjects had a single cerebrovascular accident and consequent hemiparesis were enrolled in accordance with the following requirements: chronic stroke (> 6 months after stroke event), Modified Ashworth Scale (MAS) for elbow and shoulder ≤ 3^61^, and no evidence of severe cognitive or language dysfunctions that would have interfered with the ability to understand instructions, *i.e*. Mini-Mental State Examination ≥ 25^62^. The sensorimotor status of each stroke subject was evaluated using the Fugl-Meyer Assessment scale^63^—Upper Extremity sections (maximum of 66). Fugl-Meyer scores of the contralesional limb ranged from 5 to 58, and it was 30.5 ± 19.1 for the whole population, indicating the selection of a stroke population with a prevalence of severe to moderate-mild upper limb impairments. All stroke subjects reported to be right-handed before the stroke occurrence. Eight stroke subjects had a right and seven had a left hemiparesis. Demographic and clinical data for each stroke subject are listed in supplementary material Table ST I. All subjects had no problems of visual integrity, i.e., they could clearly see the information—target and cursor positions—that was displayed in the computer screen. All control subjects were right-handed according to the Edinburgh Medical Research Council handedness scale^64^. The control and stroke subjects were equivalent in terms of age (F(1,28) = 0.03, p = 0.86) and gender. All subjects were able to apply 15 N force, i.e., individuals’ strength capacity was comfortably beyond 10 N.

The study was approved by the local Ethical Committee (Comitato Etico ASL3 Genovese, 09–04-2013, REGISTRO ASL 13/13) and conformed to the ethical standards of the 1964 Declaration of Helsinki. Each subject provided written informed consent to participate in the study and to publish individual data.

### Experimental set-up

Subjects sat on a chair holding a handle connected to a force sensor Gamma SI 13,010 (ATI Industrial 459 Automation Inc.) that was locked in a fixed position. Their forearm rested on a custom-made anti-gravity support that also prevented motion by means of suitable holders (Fig. 1A). Subjects controlled the movement of a cursor on the screen by exerting isometric forces with their hand. They had to reach eight equi-spaced targets presented one at the time on an imaginary circle (Fig. 1 B) at 14 cm distance from the central position corresponding a force of 10 N (scale factor: 1 N force = 1.4 cm cursor position shift). A 19″ LCD computer screen was placed vertically in front of the subjects, about 1 m away, at their eye level. When the cursor was at the home target, the subjects’ elbow was flexed ~ 90° and the hand position was aligned with the subjects’ midline. Targets were displayed as round green circles (1 cm radius) against a black background. Each target was presented five times (5 × 8 = 40 force exertions) in pseudo-random order: each target could be presented again only after all eight targets had been reached. The current position of the cursor was continuously displayed, as a yellow circle (0.5 cm radius), during the execution of the task; see Fig. 1A,B. In each trial, the target appeared only when the subjects were in the central target position. When the subjects reached the target, the target disappeared after that the cursor stayed inside the target for 5 s, and a new target appeared. Stroke subjects started the experiment with their contralesional arm, and their correspondent control subject started the experiment with the same arm (left or right). Subjects were asked to reach the targets as accurately as possible, without time constraints. Thus, they performed the task at their self-selected speed.

Muscle activity was recorded with surface electrodes for electromyography (CometaWavePlus wireless EMG system, Cometa Srl, Milano, Italy). Surface EMG signals were recorded from the following 16 muscles of the arm: Triceps Brachii long head (TB-long), triceps Brachii lateral head (TB-lat),Biceps Brachii short head (BB-short), Biceps Brachii long head (BB-long), brachioradialis (BRAD), pronator teres (PRON), infraspinatus (INFR), latissimus dorsi (LAT), upper trapezius (TRAP), rhomboid major (RHOM), pectoralis major (PECT),anterior deltoid (DELT-ant), medial deltoid (DELT-mid), posterior deltoid (DELT-post)., extensor carpi radialis (EXTE) and flexor carpi radialis (FLEX). Electrodes were placed according to guidelines of the Surface Electromyography for the Non-Invasive Assessment of Muscles European Community project -SENIAM^65^—and Anatomical guideline^66^. EMG electrode placement was performed according to recommendations for minimizing cross-talk from adjacent muscles^65^. Also, we verified through visual inspection of the EMG signals while performing suitable movements at the moment of the electrode placement to minimize cross-talk among muscles. Subjects were allowed to rest whenever and as long they needed. The experimental sessions lasted less than 30 min including pauses.

### Behavioral parameters

Force signals were acquired at 60 Hz. The *x* and *y* components were smoothed with a 6^th^ order Savitzky-Golay filter with cut-off frequency: ~ 8 Hz and the same filter was used to estimate the first-, second-, and third-time derivatives of the force profiles.

A trial consisted of three phases (i) reaching, where subjects, starting from the rest condition (home target—force 0 N), applied 10 N force reaching with the cursor a peripheral target; (ii) holding where subjects maintained 10 N force for 5 s, keeping the cursor inside the target; (iii) releasing, where the subjects released the force, going back with the cursor to the central target (corresponding to 0 N force).

The first five movement indicators were computed on the reaching phase. In that phase, the force onset was defined as the first instant the first-time derivative of the force exceeded the 10% of its maximum^67^. The reaching phase ended when the cursor was inside the peripheral target and the first-time derivative of the force remained under the same threshold. We considered the force applied by the end between these two-time points and we computed the following indicators:

- Average value of the first-time derivative of the force transformed into the corresponding cursor-speed units (m/s).
- Jerk index (adimensional): the square root of the jerk (norm of the third time derivative of the cursor movement), averaged over the entire cursor movement duration and normalized with respect to duration and path length^68^.
- Aspect ratio (adimensional): the maximum lateral distance from a straight line joining the start and end point of the cursor trajectory divided by the nominal distance between these two points. It is a measure of movement curvature^69^.

We also computed:

- 100-ms aiming error (deg): the angular difference between the target direction and the actual force (cursor) trajectory direction, estimated in the first 100 ms of the force exertion^70^.
- End-point error (m): the distance between the target position and cursor position when in each trial the first derivative of the force felt for the first time^71^ below 10% of its maximum.
- Force decay (N): the time required for the force to decrease by one-half with respect to its target value (5 N).

### EMG pre-processing

EMG signals were acquired at 2 kHz, band-pass filtered (30–550 Hz), rectified, low-pass filtered (cutoff: 10 Hz) to obtain the EMG envelopes^48^. To correct the inter-arm EMG-amplitude differences due to electrode placement and to ensure that the extraction of the synergies would not be biased against the low-amplitude muscles, the envelope of each muscle signal was normalized by its median value obtained overall repetitions. The normalization based on the median value instead of the maximum is more robust against high-amplitude spikes arising from noise^32^.

The normalized EMG envelope for each subject, arm and repetition was segmented in the eight target directions. For each trial we considered a time window starting 250-ms before the cursor movement onset and ending when the cursor was back to the central target, i.e., we considered all three phases, namely reaching, holding and releasing.

The normalized EMG envelope of each muscle related to each repetition, direction, task, arm, and subject was resampled on 100 time points^58^.

We calculated the Pearson correlation coefficient (ρ_EMG-INTER-GROUP_) to compare the difference in the modulation of EMG data, *i.e.* waveforms^52,72^ (ρ_EMG_-_INTRA-GROUP_)^26^. In the same way, we estimated the similarity between arms (ρ_EMG-ARM_) within groups for each muscle^20,57^.

### Spinal maps

Pre-processed EMG signals were used to estimate the motoneuronal activity in the spinal cord^21,24–27,40–42,73^ to investigate the overall muscle activity in the upper extremities, as previously described in literature^26^. To characterize the spinal motor output, EMG-activity was mapped onto the estimated location of motoneuronal-pools innervating the different muscles of the upper limb as reported by Kendall^74^. Accordingly, the activity of each spinal segment of the map was obtained as the weighted summation of the EMG activity of those muscles innervated by that spinal segment. The weight for each muscle used in the summation and the muscles innervated by each segment are reported in Table S2 in the supplementary material. The map is limited to levels between C2 and T1 in relation to the set of recorded muscles.

The metric adopted to describe the similarity between two different spinal maps was the 2D Pearson’s correlation coefficient (ρ_2D_)^26,27,42^. This metric assesses the similarity of the spatio-temporal organization of two spinal maps, i.e., the temporal changes of the muscles representing the motoneuronal activity in the spinal cord, but not their absolute intensity or level of activity. More specifically, we computed the 2D Pearson’s correlation coefficient between each stroke subject and the matched control. This was done for each arm, and we considered the average value across subjects as representative of the degree of similarity between the stroke and control subjects (ρ_2D-INTER-GROUP_). To obtain a reference value for the degree of similarity, the 2D Pearson’s correlation coefficient was computed among controls within the same arm and then averaged across individuals (ρ_2D-INTRA-GROUP_). Analogously, we estimated the similarity between the two arms (ρ_2D-ARM_) for each group.

### Muscle synergies

For each subject and arm, we extracted muscle synergies from a matrix obtained by concatenating the normalized EMG envelopes related to the eight directions averaged over the five repetitions, by using the non-negative matrix factorization (NNMF) algorithm^33^. The NNMF algorithm decomposes the normalized EMG envelopes in a defined number of positive components, or muscle synergies, each composed by an activation coefficient (H) and a vector of weight coefficients (W): the first (H) represents the timing of activity of each muscle synergy, and the second (W) the participation of each muscle in each synergy. Since, the iterative algorithm can find a solution as a local and not global minimum, the extraction was repeated fifty times and the solution explaining the highest overall amount of variance was selected^7,20,57,58^.

The number of muscle synergies has been determined as the minimum number to capture the structural variation of the original muscle activation dataset, so that, by adding one more synergy, it will only add noise to the reconstructed dataset^75^. Accordingly, for each subject, we used the common or the higher number obtained from two different methods based on the inspection of the R^2^ curve that represents the fraction of total variation explained by the synergy model^76^. The first method estimated the minimum number of synergies that achieved a R^2^ > 90%^75^. The second method was based on the detection of a change in the slope of the R^2^ curve. For the second method, a series of linear regressions were performed on the portions of the curve included between the N-synergy (N = 1 to 16) and its last point (i.e., 16th synergy). N was then selected as the minimum value for which the mean squared error of the linear regression was less than 10^−4^. In case of mismatch between the two criteria, the larger N was chosen^76^.

To simplify the comparison of H and W among subjects, the same number of muscle synergies was retained within the same arm and group; the number was established as the rounded average across subjects^58^. The NNMF algorithm does not extract muscle synergies in the same order for each subject, arm, and group. Therefore, to compare them among subjects, between arms and groups, they were ordered according to the similarity of their structure provided by the weight coefficients. For each set of synergies, the weight coefficients were ordered according to their matching with a set of reference weight coefficients by using the highest normalized scalar product between the two vectors^34^, *i.e.* the scalar products of the two vectors normalized by their norm.

The following steps describe how we obtained the reference set of weight coefficients. Since we observed that the number of muscle synergies was equal between arms and groups, we created a set of reference muscle synergies for each arm, first by pulling together the weight coefficients related to right and left arm of all control subjects, then, according to Cheung et al.^48^, we used a hierarchical clustering procedure based on the minimization of the Minkowski distance between vectors to categorize them. The number of clusters was equal to the number of extracted muscle synergies. We obtained the set of reference weight coefficients by averaging the vectors within each cluster. Then, we ordered the muscle synergies of each subject of the two groups in each arm, with respect to the similarity between their weight coefficients to the set of reference weight coefficients for that arm.

To assess the amount of variability for each synergy in each arm between groups, we calculated the cumulative or global variance^42,49,77^ explained by each synergy for each subject and arm. For each arm and synergy, we calculated the average of the cumulative variance explained.

To assess the similarity of the weight coefficients of both the contralesional and the ipsilesional arm of stroke survivors with the corresponding arm of their control subjects, we computed the scalar product (DOT_INTER-GROUP_) between the weight coefficients of the contralesional (or ipsilesional) arm of stroke subject and the corresponding arm of their controls. Then we calculated the mean values across subjects^7,20,52,57^. In the same way, we estimated the similarity between arms (DOT_ARM_) within groups.

Similarly, to assess the similarity of the activation profiles of both the contralesional and the ipsilesional arm (considered separately) of stroke survivors with the corresponding arm of their control subjects, we computed the Pearson correlation coefficient (r_INTER-GROUP_) to compare the difference in the modulation of the activation profiles (*i.e.* waveforms) between each stroke subject and the relative age-matched control for each arm, and then we calculated the mean values across subjects. To obtain a reference value to assess the degree of similarity between groups, for each arm (i.e., considering separately right and left arm), the activation profiles of each control subject were compared with the activation profiles of all other controls and then averaged across individuals (r_INTRA-GROUP_)^7,20,57^. In the same way, we estimated the similarity between arms (r_ARM_) within groups^7,20,57^.

### Statistical analysis

To test if the indicators related to behavioral performance, the number of muscle synergies, and the cumulative variance for each synergy differed between groups and arms, we ran a repeated-measures ANOVA with one within-subjects’ factor: “arm” (i.e., contralesional and ipsilesional arm for the stroke subjects and the non-dominant and dominant arm for controls); and one between groups factor, “disease” (control and stroke).

For the muscle activation patterns (ρ_EMG-GRPUP_ and ρ_2D-GROUP_), to investigate differences in force movements we added another within-subjects’ factor, the “target direction” (8 directions: 0°, 45°, 90°, 135°, 180°, 235°, 270°, 315°) in the repeated-measures ANOVA.

For the muscle synergy parameters, to investigate specific differences in synergies we added another within-subjects’ factor, the “synergy” (e.g., synergies 1, 2, 3, 4) in the repeated-measures ANOVA.

Furthermore, to investigate if the indicators of similarity between the two arms of each subject in terms of muscle activation (ρ_EMG-ARM_), spinal maps (ρ_2D-ARM_) and muscle synergies (DOT_ARM_, r_ARM_) differed among groups, we ran an ANOVA with one between groups factor, “disease” (control and stroke).

Before any ANOVA, we adopted the Greenhouse–Geisser correction when Mauchly’s test indicated that the assumption of sphericity was violated. A post-hoc analysis (Tukey’s HSD test) was used to verify statistically significant differences among factors after repeated measures ANOVA.

Bonferroni correction for multiple comparisons was applied in all post-hoc tests and not to the other main analysis^78,79^. Statistical significance was determined at the 0.05 threshold in all tests. The statistical analysis was computed within Statsoft environment (Statistica software 7.1, Statsoft TULSA, USA).

## Supplementary Information

Supplementary Information.
