# Supplementary material for: Stroke impairs the control of isometric forces and muscle activations in the ipsilesional arm
Source: Sci Rep. 2021 Sep 17;11:18533. doi: 10.1038/s41598-021-96329-0 (PMC8448776; doi:10.1038/s41598-021-96329-0)
Supplement: Supplementary file 1 — Supplementary Information. [file 41598_2021_96329_MOESM1_ESM.docx]

Stroke impairs the control of isometric forces and muscle activations in the ipsilesional arm

**Laura Pellegrino^1+^, Martina Coscia^2,3+^, Psiche Giannoni^1^, Lucio Marinelli^4,5^, and Maura Casadio^1*^**

*^1^Dept. Informatics, Bioengineering, Robotics and Systems Engineering, University of Genoa, Italy*

*^2^ Bertarelli Foundation Chair in Translational Neuroengineering, Ecole Polytechnique Federale de Lausanne, Lausanne, Switzerland*

*^3^ Wyss Center for Bio- and Neuroengineering, Geneva, Switzerland*

*^4^ Division of Clinical Neurophysiology, Department of Neuroscience, IRCCS Ospedale Policlinico San Martino, Genoa, Italy*

*^5^ Department of Neuroscience, Rehabilitation, Ophthalmology, Genetics, Maternal and Child Health, University of Genoa, Italy*

*^+^Equal contribution*

***Corresponding author:**

**Maura Casadio**

**Dept. Informatics, Bioengineering, Robotics and Systems Engineering, University of Genoa**

**Via Opera Pia 13**

**16145 Genoa, ITALY**

**Phone: +39 010 35352749**

**E-mail:** [**maura.casadio@unige.it**](mailto:maura.casadio@unige.it)

**Supplementary material Table I (ST I):** Clinical data for the stroke participants.

|  |  |  |  |  |  | FMA-UE | | MAS  (0-4) | |
| --- | --- | --- | --- | --- | --- | --- | --- | --- | --- |
|  | G | PH | E | AGE (ys) | DD (ys) | motor  (0-66) | sensory  (0-12) | Shoulder | Elbow |
|  |  |  |  |  |  |  |  |  |  |
| S01 | F | R | I | 40 | 10 | 22 | 10 | 0 | 1 |
| S02 | F | L | H | 64 | 10 | 17 | 2 | 2 | 1 |
| S03 | M | R | I | 68 | 2 | 25 | 12 | 1.5 | 2 |
| S04 | F | L | H | 57 | 1,9 | 57 | 12 | 2 | 0 |
| S05 | M | R | H | 67 | 27 | 5 | 2 | 2 | 3 |
| S06 | M | R | I | 55 | 2 | 20 | 5 | 0 | 0 |
| S07 | M | R | I | 78 | 8 | 33 | 12 | 1.5 | 1.5 |
| S08 | M | L | I | 48 | 1 | 19 | 7 | 3 | 3 |
| S09 | F | R | I | 68 | 12 | 57 | 11 | 1 | 0 |
| S10 | M | R | I | 59 | 2 | 63 | 12 | 0 | 0 |
| S11 | M | L | H | 50 | 2 | 26 | 4 | 2 | 1 |
| S12 | F | L | H | 74 | 3,5 | 32 | 0 | 2 | 2 |
| S13 | M | R | I | 59 | 0,6 | 16 | 10 | 2 | 3 |
| S14 | M | L | H | 69 | 2 | 8 | 4 | 2 | 3 |
| S15 | M | L | I | 63 | 11 | 58 | 12 | 2 | 1.5 |
|  | 5F/  10M | 8R/  7L | 6H/  9I | 61±10 | 6±7 | 30.5±19.1 | 7.7±4.4 | 1.5±0.9 | 1.5±1.1 |

G=Gender: Female/Male; PH=Paretic hand: Right/Left; E=Etiology: Ischemic/Hemorrhagic; DD=disease duration (years); FMA= Fugl-Meyer Assessment; UE =upper extremity, motor and sensory assessment; MAS = Modified Ashworth Scale 0 = normal function; 4 = severe spasticity.

**Supplementary material Table II (ST II):** Kendall’s chart for the computation of spinal maps.

|  | DELT-ant | PECT | LAT | INFR | RHOM | BB-short | BB-long | PRON | BRAD | TB-lat | TB-short | DELT-mid | DELT-post | TRAP | FLEX | EXTE |
| --- | --- | --- | --- | --- | --- | --- | --- | --- | --- | --- | --- | --- | --- | --- | --- | --- |
| C2 |  |  |  |  |  |  |  |  |  |  |  |  |  | X |  |  |
| C3 |  |  |  |  |  |  |  |  |  |  |  |  |  | X |  |  |
| C4 |  |  |  | x | x |  |  |  |  |  |  |  |  | X |  |  |
| C5 | X | X |  | X | X | X | X |  | X |  |  | X | X |  |  | x |
| C6 | X | X | X | X |  | X | X | X | X | x | x | X | X |  | X | X |
| C7 |  | X | X |  |  |  |  | X |  | X | X |  |  |  | X | X |
| C8 |  | X | X |  |  |  |  |  |  | X | X |  |  |  | x | x |
| T1 |  | X |  |  |  |  |  |  |  | x | x |  |  |  |  |  |

X corresponds to a weight coefficient of 1 and x corresponds to a weight coefficient of 0.5.

**Supplementary material Table III (ST III):** Number of muscle synergies of the stroke participants in the ipsilesional (IL) and contralesional (CL) arm.

|  | ***S01*** | ***S02*** | ***S03*** | ***S04*** | ***S05*** | ***S06*** | ***S07*** | ***S08*** | ***S09*** | ***S10*** | ***S11*** | ***S12*** | ***S13*** | ***S14*** | ***S15*** |
| --- | --- | --- | --- | --- | --- | --- | --- | --- | --- | --- | --- | --- | --- | --- | --- |
| ***CL*** | 3 | 3 | 4 | 5 | 3 | 3 | 3 | 3 | 4 | 3 | 4 | 5 | 3 | 3 | 4 |
| ***IL*** | 4 | 3 | 5 | 4 | 3 | 4 | 4 | 4 | 4 | 4 | 5 | 5 | 3 | 4 | 4 |

**Supplementary material Table IV (ST IV):** Behavioral indicators. Indicators 1-5 are referred to the cursor trajectories in the reaching phase, 6 is computed on the release phase.

| **Parameter** | **Unit** |
| --- | --- |
| 1. Average speed | m/s |
| 1. Jerk index | adimensional |
| 1. Aspect ratio | adimensional |
| 1. 100 ms-aiming error | deg |
| 1. End-point error | m |
| 1. Force decay | s |


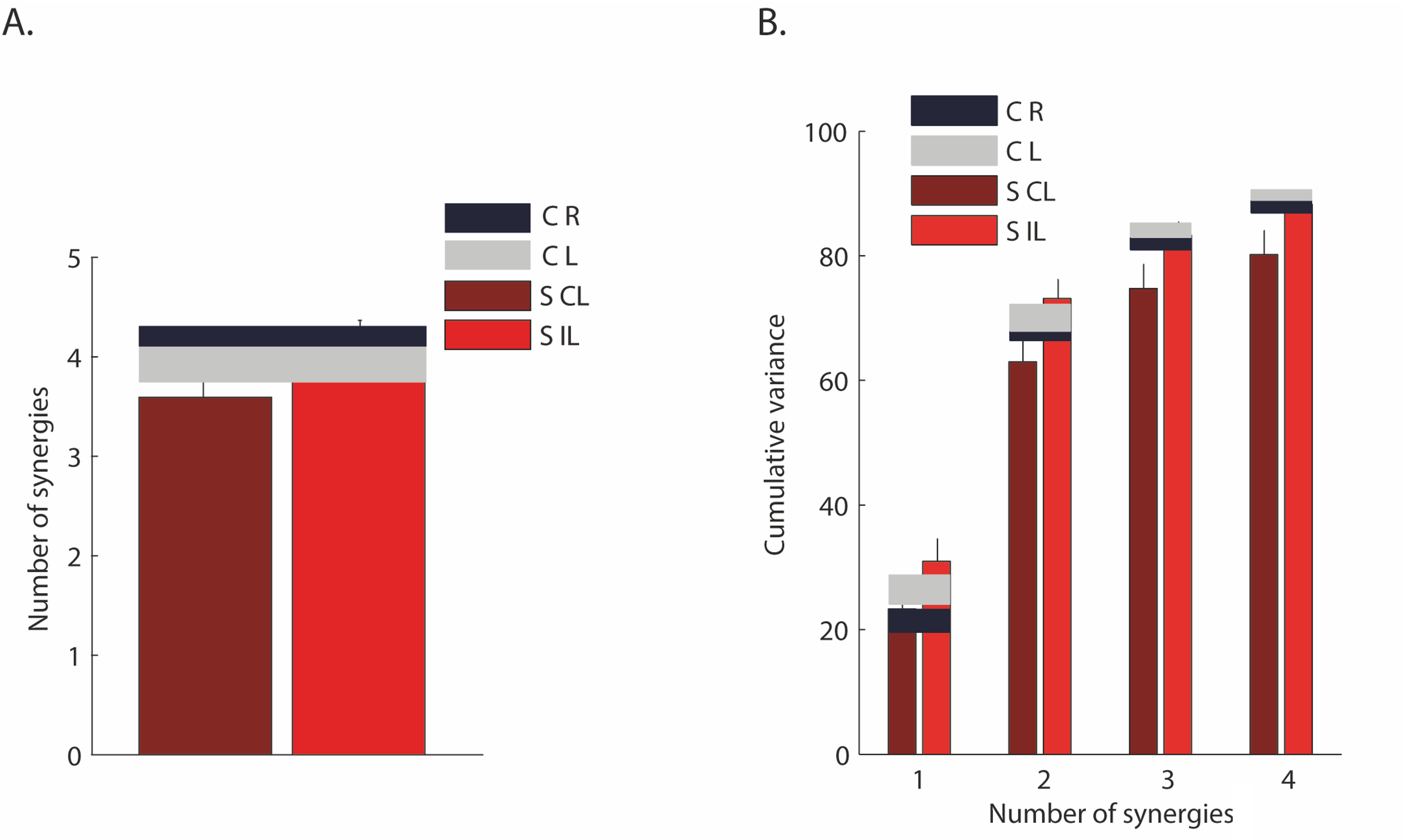


**Figure S1.** Mean and standard error of the number of muscle synergies (panel A) and cumulative variance (panel B). Darker and brighter colors represent the right (R) and left (L) arm respectively in the control subjects (C), and the contralesional (CL) and ipsilesional (IL) arm in the stroke participants (S). The error bars indicate the standard error of the indicators.


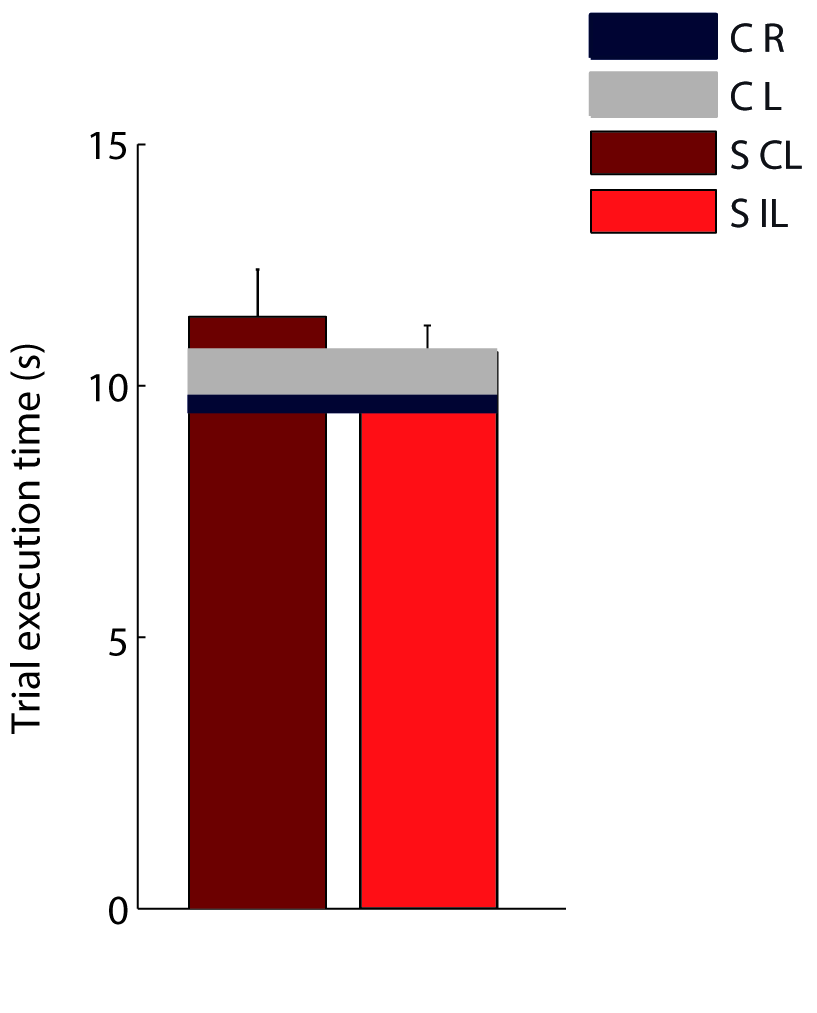


**Figure S2. Trial execution time (s).** A trial consists of three phases (i) reaching, where subjects, starting from the rest condition (home target – force 0N), applied 10 N force reaching with the cursor a peripheral target; (ii) holding, where subjects maintained 10N force for 5s, keeping the cursor inside the target; (iii) releasing, where the subjects release the force, going back with the cursor to the central target (corresponding to 0N force).

Control subjects (C) and stroke subjects (S) are shown with different colors as indicated in the legend. Darker and brighter colors represent the right (R) and left (L) arm respectively in the control subjects, and contralesional (CL) and ipsilesional (IL) arm in the stroke subjects. The error bars indicate the standard error of the indicators.


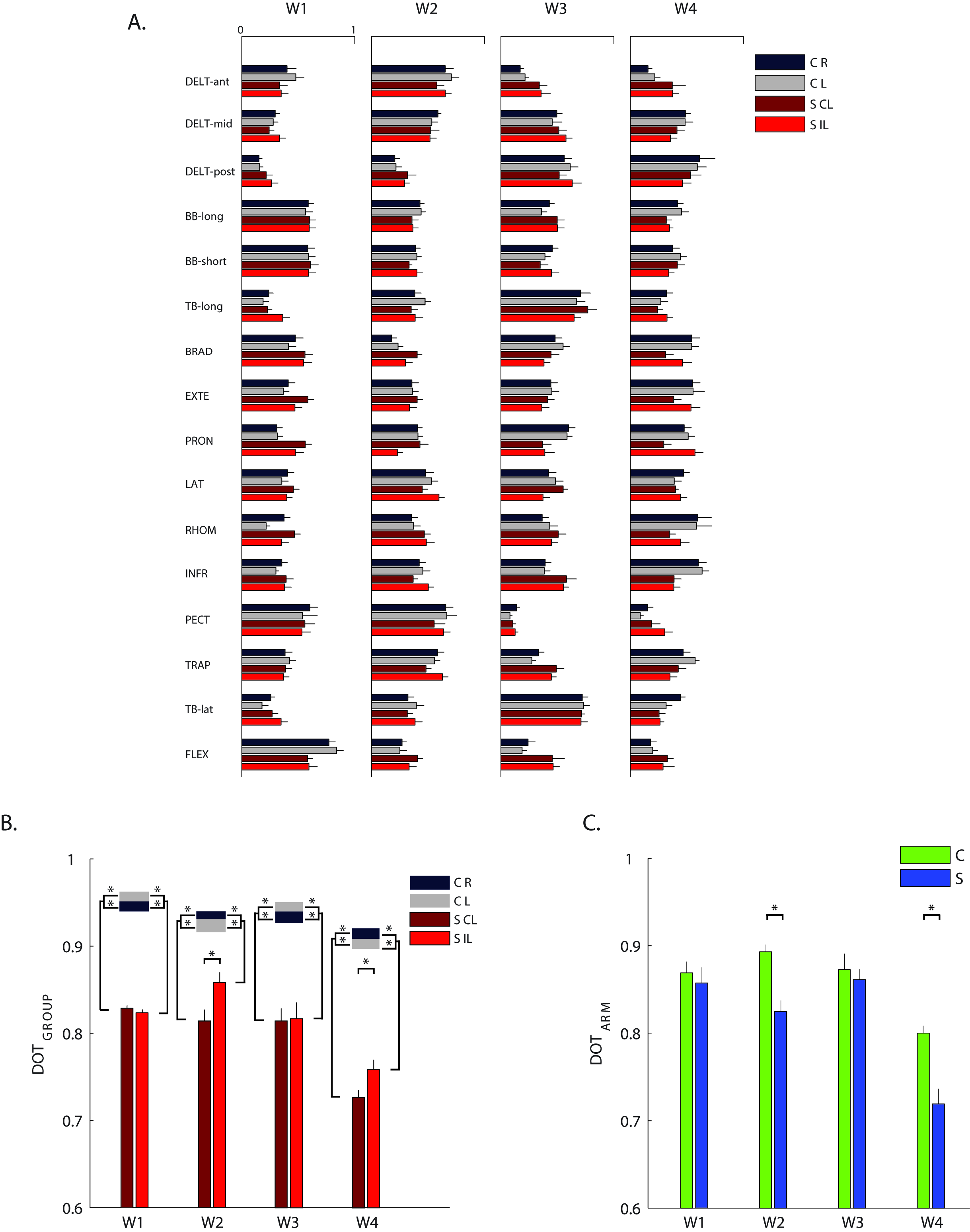


**Figure S3.** **Weight coefficients of the muscle synergies, computed during the holding phase.** Panel A: Weight coefficients for the four muscle synergies (W1 to W4). Weight coefficients were computed considering all trials, i.e., including all directions and repetitions. Control subjects (C) and stroke subjects (S) are shown with different colors as indicated in the legend. Darker and brighter colors represent the right (R) and left (L) arm respectively for the control subjects and contralesional (CL) and ipsilesional (IL) arm in the stroke subjects. The error bars represent the standard error.

Panel B: **Comparison between groups** (i.e., between stroke and control subjects) by the scalar product of weight coefficients of each muscle synergies (DOT_INTER-GROUP_). For the weight coefficient of each synergy, W1 to W4, darker and lighter red bars indicate the values obtained by comparing respectively the contralesional (CL) and ipsilesional (IL) arm of each stroke subject with the corresponding arm of his/her control subject, then averaging across the stroke group. The grey and black horizontal bars indicate the values obtained by comparing respectively the weight coefficients of the muscle synergies for the left (L) and the right (R) arms of one control subject with the corresponding arm of all the other controls, and then averaging across the control group (DOT_INTRA-GROUP_). The error bars correspond to the standard error.

Panel C: **Comparison between the two arms of a same subject** by the scalar product of weight coefficients of the muscle synergies (DOT_ARM_). For the weight coefficient of each synergy, W1 to W4, blue and green bars indicated the values obtained by comparing the two arms of stroke subjects and controls, respectively. Panel B&C: * indicate significant differences (p<0.05) between stroke and control groups.


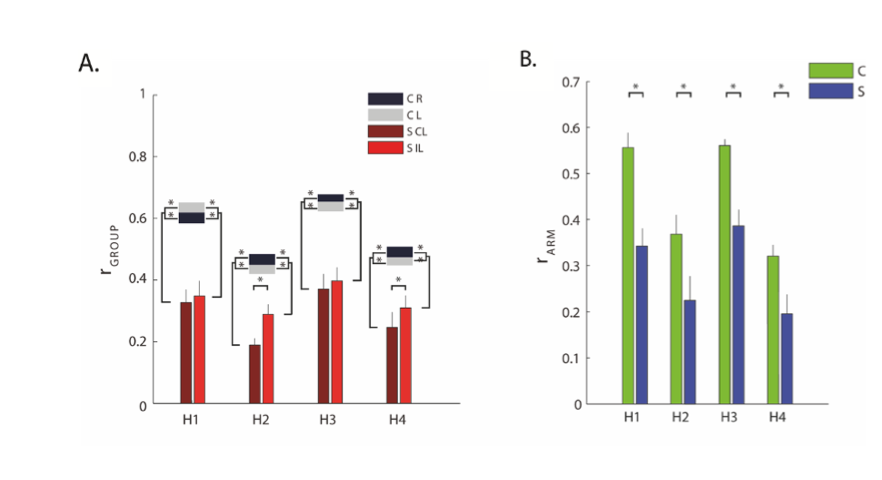


**Figure S4. Activation coefficients** (H1 to H4) **of the muscle synergies in holding phase**. Activation coefficients were computed considering all trials, i.e., including all directions and repetitions.

**Panel A:** **Comparison between groups** (i.e., between stroke, S, and control subjects, C) by Pearson correlation (r) of the activation profile coefficients of the muscle synergies (r_INTER-GROUP_). For the activation coefficient of each synergy, H1 to H4, darker and lighter red bars indicated the values obtained by comparing respectively the contralesional (CL) and ipsilesional (IL) arm of each stroke subject with the corresponding arm of his/her control subject, then averaging across the stroke group. The grey and black horizontal bars indicate the values obtained by comparing respectively the activation profiles of the muscle synergies for left (L) and the right (R) arms of one control subject with the corresponding arm of all the other controls, and then averaging across the control group (r_INTRA-GROUP_). The error bars correspond to the standard error. **Panel B:** **Comparison between the two arms of each subject** by Pearson correlation (r) of the activation profile coefficients of the muscle synergies (r_ARM_). For the activation coefficients of each synergy, H1 to H4, blue bars indicated the average value obtained for the stroke subjects (S) and the green bars the average values obtained for the control subjects (C). Panel A&B: * indicates significant differences (p<0.05) between stroke and control groups.


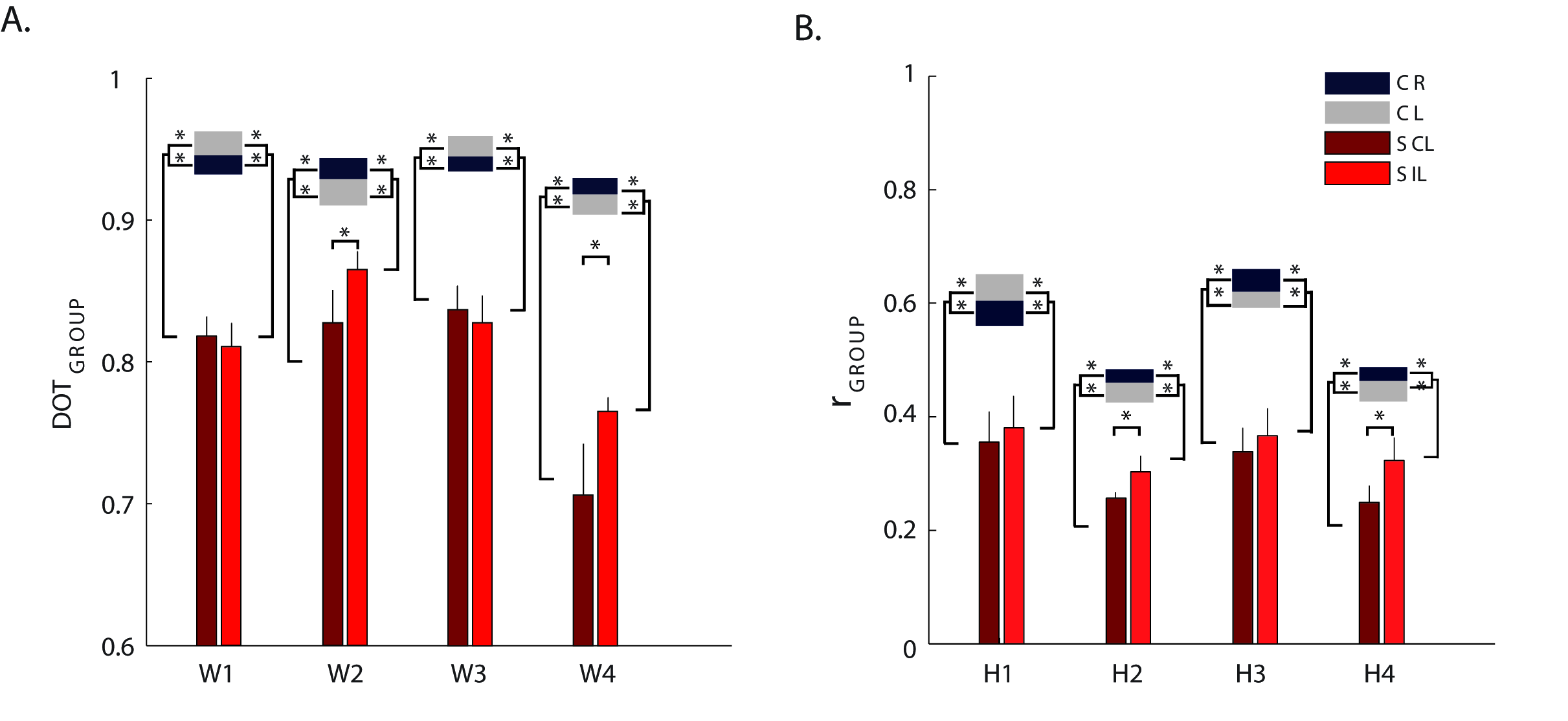
 **Figure S5. Comparison between arms of weight and activation coefficients of the muscle synergies.**

Panel A: **Comparison between groups** -i.e., between stroke (S) and control (C) subjects - by the scalar product of weight coefficients of each muscle synergies (DOT_INTER-GROUP_). For the weight coefficient of each synergy, W1 to W4, darker and lighter red bars indicate the values obtained by comparing respectively the contralesional (CL) and ipsilesional (IL) arm of each stroke subject with the corresponding arm of all control subjects, then averaging across the stroke group. The grey and black horizontal bars indicate the values obtained by comparing respectively the weight coefficients of the muscle synergies for the left (L) and the right (R) arms of one control subject with the corresponding arm of all the other controls, and then averaging across the control group (DOT_INTRA-GROUP_).

Panel B: **Comparison between groups** (i.e. between stroke and control subjects) by Pearson correlation (r) of the activation profile coefficients of the muscle synergies (r_INTER-GROUP_). For the activation coefficient of each synergy, H1 to H4, darker and lighter red bars indicated the values obtained by comparing respectively the contralesional (CL) and ipsilesional (IL) arm of each stroke subject with the corresponding arm of all control subjects, then averaging across the stroke group. The grey and black horizontal bars indicate the values obtained by comparing respectively the activation profiles of the muscle synergies for left (L) and the right (R) arms of one control subject with the corresponding arm of all the other controls, and then averaging across the control group (r_IINTRA-GROUP_).

The error bars correspond to the standard error. * indicates significant differences (p<0.05).
